# Zeissl’s Mode of Introducing Liquids into the Male Bladder without the Use of the Catheter

**Published:** 1875-06-01

**Authors:** A. Rose

**Affiliations:** New York


### ZEISSL'S MODE OF INTRODUCING LIQUIDS INTO THE MALE BLADDER WITHOUT THE USE OF THE CATHETER.

By A. Rose, M.D., New York.

*From New York Medical Record, May 8th*, 1875.

As the use of the catheter in injecting liquids into the bladder does harm to the mucous membrane in hæmorrhage, a new method has been invented by Dr. H. Zeissl, of Vienna (*Wiener Med. Wochenschrift*, 1874, Nos. 51 & 52). He simply fits the point of an irrigator into the mouth of the urethra, while the patient is placed on his back with raised nates, the penis being stretched along the abdominal walls.

I have employed this method on one of my patients under the following circumstances: M. K., 60 years old, having retired from active business, and leading a life of ease, is the father of a healthy and numerous family, having always been healthy, with the exception of a gonorrhœa contracted forty years ago, was seen by me for the first time on March 22d, 1875. The patient complains for two weeks of frequent and painful micturition. The urine is turbid, and contains a large quantity of muco-purulent sediment. He is feverish, has neither sleep nor appetite. I tried to enter the bladder with a double catheter, but did not succeed on account of strictures. I then tried dilatation, beginning with a No. 6 steel sound, and finally succeeded in introducing No. 12. The first dilatations were painful, and followed by chills. In spite of the administration of quinine and the hypodermic use of morphine, the patient claimed to have these chills still. He refused to let me make an incision into the strictures. On April 8th I introduced a double catheter, No. 10, which I connected with a fountain syringe filled with tepid water. Mucus and blood coagula filled one canula to such an extent that while the water entered freely, it could return only through one channel. During and after the introduction of the catheter there was great pain and fever. I saw the patient again on April 12th. The urine was clearer, and but little sediment was found. For internal use I had ordered the bicarbonate of soda, and lime water with milk, and Vichy water to be used alternately. Want of appetite and sleep still existed.

Having in the meantime read of Zeissl's method, I introduced a glass tube into the urethra, having first connected it with the India-rubber tube of a fountain syringe which contained about eight ounces of a tepid solution of tannin, which I allowed to pass into the urethra from a height of four feet. The patient experiences, after half a minute, while the liquid entered the bladder, a feeling of comfort, just as described by Zeissl's patients. I removed the glass tube as soon as the liquid began flowing out of the urethra alongside of the tube. The patient, feeling a desire to pass the water, emptied a liquid containing mucus and tannin.

April 13th. There is a decided improvement in every respect; the urine is nearly clear, there being but a very small quantity of sediment. The patient has been able to sleep a few hours.

April 14th. Renewed introduction of solution of tannin into the bladder.

April 15th. Patient has slept better than ever since he took sick; has no fever; his appetite is returning, and he feels altogether encouraged. Steel sound No. 12 passed for the first time with ease and without leaving any pain.

Foreign Bodies in the Digestive Canal.—The incident of the *homme a la fourchette*, the man who swallowed a fork in Paris in April last, has inspired Dr. Mignon with the idea of collecting all records of similar cases. He has been able to find details of one hundred and sixty-three, and it would be difficult to imagine anything more astonishing than the catalogue (given in the *Union Medicale* for Nov. 3) of the objects swallowed by either veritable lunatics or what may be termed sane idiots. Amongst the very indigestible and uncomfortable items catalogued, we find fifteen gold medals, hair rings innumerable, 175 francs, a shoe buckle, nine inches of a sword blade, very sharp scissors, eighty pins, a baby's bottle, the castor of a night-stool, an entire set of dominoes (the size of which however is not stated), one hundred *louis d'or*, a flute four inches long, a glass phial, thirty-five knives, a clay pipe, from fourteen to fifteen hundred pins, a bar of lead weighing a pound, a whetstone, and (in three instances) a table fork. But the most extraordinary of all these cases occurred in the instance of a convict who died in Brest, in 1773, and on whose body a necropsy was performed. The stomach was completely displaced and occupied the left hypochondrium, the lumbar and iliac regions of the one side extending into the pelvis nearly as far as the foramen ovale; it contained fifty-two different objects, weighing altogether one pound ten ounces. Amongst them was a part of the hoop of a barrel, nineteen inches long and one wide. M. Mignon has classified these 163 cases into three categories: 1. Foreign bodies which passed through the whole extent of the digestive canal with scarcely any injurious results. 2. Foreign bodies which have passed through the whole extent of the digestive track, with more or less serious results, but ultimate recovery. 3. Foreign bodies which have passed through the whole digestive track, causing serious disturbance and fatal results. 4. Cases in which the foreign body has not been passed. 5. Cases in which operations have been performed. It is a remarkable fact that the cases of death caused by the presence of foreign bodies in the digestive tubes are less numerous than might be expected. Out of the 163 cases, we only find ten deaths from this cause. To these must be added two deaths after operation, making altogether twelve, or 7.3 per cent. There appears, therefore, to be no great cause for the surgeon to be over-anxious in these cases, but to remember, that, unless there should either be some complications in the general health or some special indication, it will be as well for him not to interfere, and above all things not to perform gastrotomy, save as a last resource. Of this last operation, M. Mignon relates five cases; amongst them being those which Mr. Neal, in 1853, and Mr. Bell, in 1859, thought themselves obliged to perform; the one in order to extract a bar of lead ten inches long, and weighing a pound, the other to do the same with a bar of lead, nearly twelve inches long, and weighing more than nine ounces. In both these cases the symptoms were very serious, comprising violent pains in the stomach, twitchings along the vertebral column, sickness, and general prostration. The foreign bodies could not be felt through the abdominal walls, but the surgeons decided on performing the operation, thinking that the sufferers had no chance of relief by expulsion *per anum*. The success of the operations was fortunately complete. — *Lond. Med. Rec.*, Dec. 2, 1874.

---

Dr. A. H. Thompson, in the *Dental Cosmos*, suggests that, as our teeth continue to deteriorate in the present day, it is probable, on the Darwinian theory, that future generations will not have any teeth developed in their jaws, as being quite superfluous when the manufacture of artificial teeth shall have reached perfection.

TREATMENT OF UNUNITED FRACTURES.—Dr. Nicoladoni, quoted in the *London Medical Record*, gives the following modification of what is known as "Dumreicher's Method:"

The injured leg, in which there is a tendency manifested to the formation of a false joint, is enveloped from the toes to a part a little below the fracture by a strong flannel bandage. Four wedge-shaped pads are applied, two above and two below the fracture, in such a manner that the broad ends of the upper and lower pairs are opposed to each other, and that between them a free surface of skin is left which corresponds to the seat of fracture. These pads are kept in position by strips of adhesive plaster, and covered by a thin wooden splint, over which a bandage is firmly applied. The whole limb is then kept at rest on an ordinary splint. By the application of the bandage below the fracture the peripheral portion of the limb is protected against the injurious results of pressure made by the pads above the ankle. The pressure of the lower pads and of the flannel bandage induces an active arterial hyperæmia of the parts about the false joint, which hyperæmia is more or less restricted to these parts, as the two pads are so applied as to retard the backward flow of venous blood, though not interfering very much with the arterial supply. After an application of this apparatus for twenty-four hours, the skin becomes red and hot, and the fractured portion of bone can no longer be felt, on account of the swelling of all the superjacent soft parts. This swelling is firm, and differs altogether from ordinary œdema. On the second and third days, the parts between the wedge-shaped pads become more swollen and firmer; but this swelling will speedily disappear on the removal of the pads; and will not persist and do any good until after the apparatus has been retained for five or six days. In favorable cases, after an application kept up, with occasional short intervals, for three or four weeks, the fragments become much less movable, and cannot be examined and moved without much pain. The limb can now be placed in a firm apparatus of plaster of Paris, or water-glass, and in a few weeks the fracture will become firmly consolidated. In the first case reported by Dr. Nicoladoni, the pads were applied during eight weeks, with occasional intervals of rest lasting for two days. A gypsum bandage was then applied, and at the end of the tenth week the fragments of tibia were found to be firmly consolidated. In the second case the pads and bandages were applied during a period of two months; six weeks after their removal and the application of a firm bandage, there was perfect union.—*Phil. Med. and Surg. Reporter*.

---

ON THE ACTION OF MERCURY, PODOPHYLLIN, AND TARAXACUM ON THE BILIARY SECRETION—In some comments in the *Practitioner* (Dec., 1874) on the report of the committee of the British Medical Association, of which Dr. Bennett was chairman, the reviewer says that the experiments made show that calomel and other mercurials, podophyllin and taraxacum, do not increase the secretion of bile in an animal having a biliary fistula. From the care with which these experiments were made, there can be little doubt of their correctness; but the opinions which Dr. Bennett founds on them, and supports with his usual force and vigor in the appendix, are quite erroneous. He completely disregards the results of clinical experience, and condemns the use of mercury as a cholagogue, because it does not increase the amount of bile poured out by the liver. He totally forgets the fact that what we want in cases of biliousness is not increased secretion by the liver, but removal of bile from the blood. As was pointed out in the June number of this journal, this result may be attained by the mercurials removing the bile from the intestine and preventing its re-absorption, quite as readily as by stimulating the liver to increased action.

TREATMENT OF DIPHTHERIA—Dr. H. V. Sweringen publishes in the Philadelphia *Medical Times*, an account of his method of treating diphtheria. He has little or no faith in local treatment, except in cases where it is absoluteny necessary to remove excessive mechanical obstruction of the air passages, or to correct the fœtor by disinfectants. The line of treatment pursued was as follows:

℞.—Quin. sulph., gr. xxxij.;
Acid. tannic., gr. x.;
Syr. simp., f ℥ ii.;
Tr. ol. menth. pip., gtt. iij.—M.

Ft. mist.

Sig.—A teaspoonful every three hours until cinchonism is induced.

After which he administers the following:

℞.—Potassi iodidi, gr. xxxij.;
Potassii bromidi, ʒ ij.;
Syr. simp.;
Tr. cinch., co., aa f ℥ i.—M.

Ft. sol.

Sig.—A teaspoonful every three or four hours.

The above may be given alternately with the following:

℞.—Tr. ferri chlor., f ʒ ij.;

Syr. simp., f ʒ vi.—M.

Ft. mist.

---

QUOT HOMINES, TOT SENTENTIÆ.—We are not quite satisfied with the result of the election of the president. It is not that we grudge Dr. Sims the honor that comes to him; he has a well-earned and world-wide reputation, and we have no doubt will do credit to the position; but we think the choice might have fallen upon others whose associations are more strictly American [or Boston?], and who are more generally looked up to as leaders of the profession.—*Boston Journal*, May 13, 1875.—*Cin. Clinic.*

---

AMERICAN MEDICAL ASSOCIATION.—By request of many members, I will shortly issue a pamphlet edition of the minutes of the Louisville Session. Those who desire copies should remit fifty cents at once, as a limited edition will be printed. WM. B. ATKINSON,
1400 Pine St., Philadelphia.

STATISTICS OF A HUNDRED CASES OF OPERATIONS FOR STONE.—Sir Henry Thompson has given a brief record of his last hundred cases of operation on the adult, excluding all cases below 22 years of age, of which there happened to be one. There are in the list only four cases below 50, sixty-five cases being above 60, and the mean age of the entire list of cases being not less than 63½ years. The first operation of this series was done shortly before Christmas of 1872, and the record represents all his work in this direction for two years and a quarter, the case of the Emperor Napoleon being the third of the series. Ninety-six were adult males, and four were adult females. Of the ninety-six males eighty-seven were operated on by lithotrity, and nine by lateral lithotomy.

The mean age of the eighty-seven operated on by lithotrity was 63½ years, the oldest being 83, the youngest 22, but only four were below 50 years.

The mean age of the nine operated on by lithotomy was 63½ years also, their ages being also between 36 and 79. Among the eighty-seven operated on by lithotrity were four deaths; the ages were 65, 65, 66, and 81. Among the nine operated on by lithotomy were two deaths, viz., at 61 and 63. Thus it is observed that there was a total of six deaths in ninety-six patients, by the two operations, with a mean age of 63½. In this series allusion is made to what may be termed an extraordinary run of luck. It was a succession of fifty-one elderly cases without a single death, these cases having a mean age of 64 years. The author states that he hopes soon to publish his experiences in an unbroken series of five hundred cases in the adult male, besides the cases of women and children.—*The Lancet*, April 3, 1875.

---

DR. E. H. TRENHOLME ,reports (*Canada Medical Record*, p. 507, 1875) a case of traumatic tetanus successfully treated with chloral and bromide of potassium.

VENEREAL ULCERS.—Dr. Godon (*Pacific Medical Journal*) publishes a series of cases of venereal ulcers treated with iodoform, and concludes his paper with the following remarks:

1. That iodoform is a therapeutic agent producing more certainly, and more promptly than all others ordinarily employed, the cicatrization of syphilitic ulceration in general.

2. That in the treatment of chancroids, iodoform is in some manner a specific by the promptitude with which it produces cicatrization, and this too without pain.

3. That iodoform in the treatment of simple or virulent buboes can be employed in the form of an ointment as a resolvent during the early stage, with more success than any other agent.

4. Iodoform acts as a topical agent and also as a local anæsthetic. That by its employment in these cases it effects a cure without producing any pain, obviating the dread of burning and the attendant pain which deter so many unfortunates from applying immediately to the surgeon for relief. That the rapid cicatrization which it causes is owing to the simplicity of the dressing, which does not irritate the diseased parts, to the absorption of the secretions by the iodoform powder, to the antiseptic properties of the medicament, and to the presence of iodine, which acts favorably on venereal ulcerations in general.

---

TREATMENT OF ANAL VEGETATIONS.—M. Cruveilhier, of the Hopital St. Louis, believes with the majority of surgeons, that these sessile or pediculated vegetations are not dependent on the syphilitic virus, and that therefore internal treatment is not appropriate. They must be destroyed locally in a radical manner, for if a single one is allowed to remain, however small it may be, it will form the starting point of new vegetations, which will multiply without measure. He has employed excision with curved scissors, followed or not by cauterization with diluted perchloride of iron. But he prefers a milder and more efficacious remedy, pure chromic acid, which, applied to the small tumors, the healthy parts being protected, mummifies them and causes their decay. This caustic is less painful than nitric acid, or acid nitrate of mercury, and less often causes inflammation in the neighborhood. If these means fail, the operation of removal with the knife must be resorted to.—*Revue de Therap.*, 15, 1874.—*The Doctor.*

---

NON-NITROGENOUS DIET. — Last year we called attention to the experiments of Prof. Parkes on the effects of an absolutely non-nitrogenous diet.

Dr. Andrew, in the new volume of "St. Bartholomew's Hospital Reports," informs us that he has submitted eight cases of acute rheumatism to this diet, which he believes promises better results in this disease than any other treatment. The pains disappeared on the average in 3.75 days; the longest time being 5, the shortest 2.

It is to be remarked, that only young patients whose powers of nutrition are unbroken, are likely to bear with benefit this powerful method of treatment; and even in these it cannot be continued very long. Dr. Parkes himself showed that the power of the heart is greatly reduced by this diet within twenty-four hours. It would seem, therefore, that such a plan is only adapted for acute cases. Nevertheless, Dr. Moss, of the Royal Naval Hospital, Esquimalt, Vancouver's Island, has applied it, though without much success, to *chronic* rheumatism, which it appears is very common in that island. The diet adopted was that given by Dr. Parkes, with the addition of tapioca pudding without milk or eggs. It consisted of arrowroot cakes made with butter, and sometimes with sugar or treacle. Dr. Moss gives the details of the weights and quantities. The bowels became regular, the rheumatic pains lessened, and dyspepsia disappeared.—*The Doctor.*

